# Three fourths of women of reproductive age in emerging regions of Ethiopia are facing problems in accessing health care

**DOI:** 10.1038/s41598-023-36223-z

**Published:** 2023-06-30

**Authors:** Samrawit Mihret Fetene, Tsegaye Gebremedhin Haile

**Affiliations:** Department of Health Systems and Policy, Institute of Public Health, College of Medicine and Health Sciences, University of Gondar, P. O. Box: 196, Gondar, Ethiopia

**Keywords:** Health services, Public health

## Abstract

Providing adequate and equal access health care is a key goal towards universal health coverage (UHC), but women continue to confront considerable inequities in accessing healthcare, particularly in the emerging regions of Ethiopia. Therefore, we identified the contributing factors to the problems in accessing health care among women of reproductive age in emerging regions of Ethiopia. Data from the 2016 Ethiopia Demographic and Health Survey were used. A total of 4680 women in reproductive age were included in the final analysis and a multilevel mixed-effect binary logistic regression analysis was done to identify the contributing factors to the problems in accessing health care. In the final model, a p-value of less than 0.05 and adjusted odds ratio (AOR) with 95% confidence interval (CI) were used to declare statistically significant factors. We found that 71.0% (95% CI 69.64–72.24%) of women in reproductive age had problems in accessing health care. Unmarried women (AOR = 1.30 95% CI 1.06–1.59), uneducated (AOR = 2.21 95% CI 1.48–3.30) and attended primary school (AOR = 1.58 95% 1.07–2.32), rural resident (AOR = 2.16 95% CI 1.40–2.02), poor (AOR = 2.95 95% CI 2.25–3.86) and middle wealth status (AOR = 1.74 95% CI 1.27–2.40), women who gave two births (AOR = 1.29 95% CI: 1.02–1.64) and not working (AOR = 1.33 95% CI 1.06, − 1.68) and working in agriculture (AOR = 1.88 95% CI 1.35–2.61) were factors that contributed for the problems in accessing health care. A significant proportion of women of reproductive age in emerging regions of Ethiopia face challenges in accessing healthcare, which places the country far from achieving its UHC targets. This issue is particularly prominent among unmarried, poor and middle wealth status, uneducated, non-working, and rural women of reproductive age. The government should develop strategies to improve women’s education, household wealth status, and occupational opportunities which would help to alleviate the barriers hindering healthcare access for women residing in emerging regions of Ethiopia.

## Introduction

The healthcare systems continue to face a challenge in providing accessible, high-quality, comprehensive and integrated care around the world^1^. Globally, the health community is setting ambitious targets for UHC, and there is an increasing interest in accessing primary health care in low-and middle-income countries^2^. Accordingly, the UHC includes: financial risk protection, access to quality essential health-care services and access to safe, effective, quality and affordable essential medicines and vaccines for all^3^, but half of the world's population lacks adequate access to basic health services^4^. Maternal health issues continue to be a major source of concern and an unfinished business on the millennium development goal agenda for Africa^5^. The maternal mortality ratio (MMR) in developing countries is 15 times higher than in developed countries^6^. Reduction of preventable maternal and newborn mortality is vital to the attainment of sustainable development goals (SDGs)^7^.

Access has been defined as the "degree of fit" between health care clients and the health care system^8,9^. Whether individuals and groups actually gain access to health services depends on issues such as the availability, accessibility, affordability, acceptability, and accommodation of services^10^. Access to healthcare has been emphasized as the major barrier towards the utilization of maternal health services in low-income countries^11,12^. Furthermore, women continue to face significant disparities in access to and utilization of healthcare^13^. The consequences of difficulty in accessing health care among women in reproductive age include unwanted pregnancies, unsafe abortion, maternal and child mortality resulting from low family planning uptakes, and home deliveries^14,15^. However, if all women had access to essential health services, an estimated 74% of maternal deaths could be avoided^16^.

Ethiopia has a significant contribution for the highest maternal mortality in Africa with 401 maternal deaths per 100,000 live births in 2017^17^ that can be explained by inaccessibility of health care. In that regard, significant work remains in order to end the millions of preventable child and maternal deaths that occur annually^18^. In order to reach the rural population in Ethiopia, the primary health care units comprised health posts, which are mainly intended to provide essential health services at the community level by health extension workers^19,20^. Subsequently, a complete set of care for priority maternal and child health interventions and infectious diseases (tuberculosis, malaria, and HIV) are provided free of charge at all public health facilities. Despite the fact that access to health care has improved, there are still significant geographic differences in health care utilization and outcomes^19^. Previous studies identified the socio-demographic and economic variables: age, educational status, resident, marital status, and wealth index were the factors contributing to problems in accessing health care^21–29^.

Currently, the health sector is providing different forms of support to communities and regions left behind, i.e., emerging regions. These regions are characterized by scattered pastoralist and semi-pastoralist societies suffering from extreme poverty^30^. Pastoralist communities in Ethiopia are generally lagging behind in major health indicators^19^. Although small numbers of studies have been conducted in different parts of Ethiopia, evidence on problems in accessing health care among reproductive age women remains limited, particularly in emerging regions. As a result, understanding contributors of problems in accessing health care in emerging regions could help health policymakers, implementors, and other key stakeholders in developing strategies to alleviate the identified problems and to promote women's health care access for each segment of the population in the country. More broadly, it allows Ethiopia's government to make substantial progress toward the UHC. Therefore, this study aimed to identify the individual and community level factors that contribute to problems in accessing health care among reproductive age women in emerging regions of Ethiopia.

## Methods

### Data sources and context

The recent 2016 Ethiopia Demographic and Health Survey (EDHS) data were used in this study, a community-based cross-sectional survey. The EDHS is a five-year national representative household survey conducted by Ethiopia's Central Statistical Agency^31^. In Ethiopia, during the survey, there are nine regional states (Tigray, Afar, Amhara, Oromia, Benishangul-Gumuz, Gambela, South Nation Nationalities and Peoples' Region (SNNPR), Harari and Somali) and two city administrations (Addis Ababa and Dire-Dawa). These nine regions can be divided into emerging regions (Afar, Benishangul-Gumuz, Gambela, and Somali) and developed regions (Tigray, Amhara, Oromia, SNNPR, and Harari). The emerging regions are characterized by scattered pastoralist and semi-pastoralist societies suffering from life-threatening poverty^30,32^.

### Population, sampling procedures, and sample size

The EDHS employed two-stage stratified sampling techniques from an existing sample frame, generally the most recent census frame, to select the study participants. In the first stage of selection, the primary sampling units were selected with a probability proportional to their size within each stratum. The primary sampling units are typically census enumeration areas (EAs). The primary sampling units forms the survey cluster. In the second stage, a complete household listing was conducted in each of the selected clusters. Following the listing of the households, a fixed number of households were selected by equal probability systematic sampling in the selected cluster. The overall selection probability for each household in the sample is the probability of selecting the cluster multiplied by the probability of selecting the household within the cluster. In the DHS, all women aged 15–49 years who are regular members of the selected households were eligible for the survey.

In our study, we included all women in reproductive age in the four regions (emerging regions). Finally, a total of 4680 women in reproductive age who reside in emerging regions were identified from the EDHS 2016 and included in this analysis.

### Variables and measurements

The dependent variable was problems in accessing health care. The problems in accessing health care among women in reproductive age were measured when a woman has serious problems in accessing health care for herself when she is sick^33^. In our study, if a woman has at least one of the four serious problems (as listed in Table 1) in accessing health care measurements, we considered as "having problems in accessing health care", otherwise "not having problems"^26,34^.

**Table 1:** Problems in accessing health care.

No.	Problems in accessing health care includes
1	Problems in getting permission to go for treatment
2	Problems in getting money for treatment
3	Problems in distance to the health facility
4	Problems in not wanting to go alone during their sickness

This study categorized independent variables into individual and community variables. Variables such as education, occupational status, religion, sex, marital status, family size, and household wealth status, birth in the last 5 years, parity, antenatal care (ANC) follow-up for recent pregnancy, institutional delivery, had at least one birth, contraceptive uses, continuum of care and postnatal care (PNC) check-up were included in this analysis as independent variables.

In order to measure household wealth status, the asset index was used based on data from the entire sample and was calculated separately for rural and urban households. All scores were combined into one asset index and ranked into three categories (poor, middle, and rich). The other categories of independent variables were community level, which included the place of residence, health insurance coverage, region, and media exposure.

### Data processing and analysis

Data were extracted, cleaned, recoded, and analyzed using STATA 16. The descriptive statistics were presented in tables, graphs, and narrations. The nature of EDHS data is hierarchical (individuals were nested within communities), which violates the independent assumptions of the standard logistic regression model. Therefore, a multilevel logistic regression analysis model was fitted to identify both the individual and community-level variables that contributed to the problems in accessing health care.

In this study, we fitted four models: (i) null model, a model without explanatory variables; (ii) model I, a model with individual-level factors; (iii) model II, a model with community-level factors; and (iv) model III, a model with both individual and community-level factors simultaneously. Since the models were nested, model comparison and fitness were checked based on the Intraclass Correlation Coefficient (ICC), Likelihood Ratio test^35^, Median Odds Ratio (MOR,) and deviance (− 2*log likelihood ratio (LLR) values. Model III was chosen as the best-fitted model i.e. model with the lowest deviance. The variation between clusters was assessed by computing ICC^36^. An ICC greater than 5% is eligible for multilevel analysis; in our analysis, the ICC was 37%.

In the bivariable analysis, variables with a p-value of < 0.2 were considered for the multivariable analysis. Finally, an AOR^23^ with 95% CI and a p-value of < 0.05 were used to declare significant factors contributing to the problems in accessing health care.

### Data quality control

Any data collection procedure, including the EDHS, has the potential to produce outliers or inaccurate data. However, the EDHS implemented various measures to minimize such occurrences and ensure the accuracy of the data. The survey used a standardized methodology and questionnaire, and data collectors received extensive training to minimize errors in data collection. In addition, field supervision and quality control measures were in place to monitor the data collection process and identify any potential issues. The EDHS also conducted data quality checks and data cleaning procedures to identify and correct errors and inconsistencies in the data. In general, the EDHS data considered rigorous quality control processes to ensure the accuracy and reliability of the data. Details on the data quality are published in the EDHS 2016 report^34^. Moreover, we followed the guide to demographic health survey (DHS) statistics^33^ to ensure data quality and handle missing data.

### Ethical considerations

The study used a secondary analysis of publicly available survey data from the DHS program (available at https://www.dhsprogram.com/Data/), and the data has no personal identifier. To conduct our study, we registered and requested the dataset from DHS online archive. Then, permission was granted to the authors by the MEASURE DHS program to use this data for this study. Data from the original EDHS were collected under international and national ethical guidelines. The Ethiopian Public Health Institute, the former Ethiopian Health and Nutrition Research Institute Review Board, the National Research Ethics Review Committee at the Ministry of Science and Technology, the Institutional Review Board for ICF Macro International and the Centers for Disease Control and Prevention provided ethical approval for the survey.

## Results

### Description of the study participants

The socio-demographic and economic characteristics of study participants are presented in Table 2. Nearly 60% of the women were not going to school. Sixty-four and 72% of women were Muslim religion followers and married, respectively. Majority (62.4%) of the women were in the poor household wealth status; 60.6% of women do not have work; and 51.8% of the household had more than four family members.

**Table 2:** Socio-demographic and economic description of reproductive age women (n = 4680).

Variables	Frequency (n)	Percentage (%)
Age in years		
15–24	1933	41.3
25–34	1529	32.7
35–49	1218	26.0
Educational status		
No education	2756	58.9
Primary	1280	27.4
Secondary	433	9.2
Higher	211	4.5
Religion		
Muslim	2997	64.0
Protestant	870	18.6
Orthodox	710	15.2
Others*	103	2.2
Current marital status		
Unmarried	1318	28.2
Married	3362	71.8
Occupational status		
Not working	2836	60.6
Professional	646	13.8
Agricultural	853	18.2
Others**	345	7.4
Sex of the household head		
Male	2984	83.8
Female	1696	36.2
Household wealth index		
Poor	2919	62.4
Middle	389	8.3
Rich	1372	29.3
Family size		
1–5	2424	51.8
5 +	2256	48.2

*Others: catholic, traditional, Job **merchant, self-employed, daily labor.

### Obstetric characteristics and reproductive health services related variables

Table 3 shows the obstetric and reproductive health related variables. Forty five percent of the women did not give birth in the last five years and 12.1% of women use modern and traditional contraceptive methods. Additionally, of the total 2563 women who were assessed for their most recent births, 52.6%, 24.9%, 7.1%, and 3.7% of them were had ANC visits, institutional delivery, PNC check-up till 2 months, and continuum of care (ANC + institutional delivery + PNC), respectively.

**Table 3:** Obstetric characteristics of reproductive age women (n = 4680).

Variables	Frequency (n)	Percentage (%)
Births in the last 5 years		
No birth	2117	45.2
One	1275	27.2
Two	1013	21.7
Three and above	275	5.9
Had at least one birth before (include live and still births)		
Yes	3397	72.6
No	1283	27.4
Parity (n = 3397)		
Primi-para	558	16.4
Multi-para	1372	40.4
Grand multi-para	1467	43.2
ANC follow-up for recent pregnancy (n = 2563)		
Yes	1347	52.6
No	1216	47.4
Institutional delivery (n = 2563)		
Yes	639	24.9
No	1924	75.1
Postnatal care/checkup (n = 2563)		
Yes	182	7.1
No	2381	92.9
Continuum of care (ANC + Delivery + PNC) (n = 2563)		
Yes	96	3.7
No	2467	96.3
Contraceptive uses		
Using modern and traditional methods	567	12.1
Non-user-intends to use later	1128	24.1
Does not intend to use	2985	63.8

### Community level factors

Majority (78.0%) of the reproductive women were residing in the rural community and less than half a percent was have health insurance coverage which are presented in Table 4.

**Table 4:** Community level variables for reproductive women (n = 4680).

Variables	Frequency (n)	Percentage (%)
Region		
Afar	1128	24.1
Somali	1391	29.7
Benishangul-Gumuz	1126	24.1
Gambela	1035	22.1
Residence		
Urban	1028	22.0
Rural	3652	78.0
Exposure to mass media		
Yes	151	3.2
No	4529	96.8
Health insurance coverage		
Yes	20	0.4
No	4660	99.6

### Problems in accessing health care among women of reproductive age

**Figure 1:**
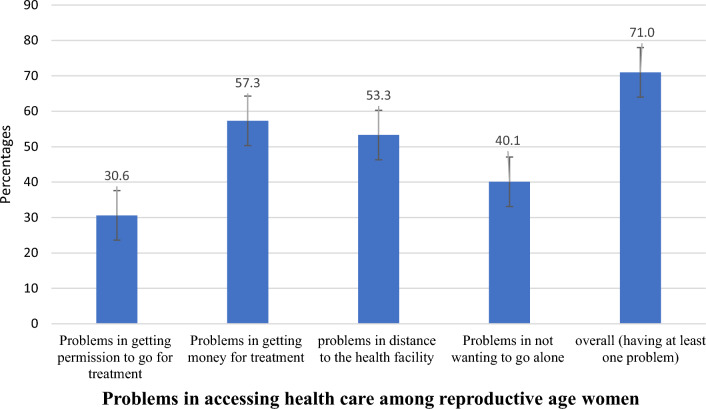
Problems in accessing health care among reproductive age women in emerging regions (n = 4680).

In our analysis, 71.0% (95% CI 69.64–72.24%) of women of reproductive age were had at least one serious problem in accessing health care in emerging regions of Ethiopia. High problem (57.3%) was observed in getting money for treatment and less (30.6%) problems were found getting permission to go for treatment which are presented in Fig. 1.

#### Measure of variation using random effects and model fitness

There was a significant variation of problems in accessing health care among women of reproductive age across the individual and community level in the emerging regions of Ethiopia. The ICC of problems in accessing health care among women of reproductive age in the null model was 0.371 (95% CI 0.312–0.432); meaning 37.1% of the variation in problems in accessing health care among women in reproductive age was due to the differences between clusters (between-cluster variation). The model fitness was checked using the ICC across the four models, Akaike Information Criterion (AIC) and deviance which are presented in Table 5. Accordingly, model 3; a model with low deviance and AIC was chosen.

**Table 5:** Random-intercept model of multilevel analysis for problems in accessing health care among women in reproductive age (n = 4680).

Measure of variations	Null model	Model 1	Model 2	Model 3
Variance				
ICC	0.371	0.254	0.269	0.237
Model fitness				
Deviance (− 2*LLR)	4848	4638	4770	4622
AIC	4852	4675	4786	4670

*AIC* Akaike information criterion, *ICC* intraclass correlation coefficient, *LLR* log likelihood ratio.

### Factors associated with problems in accessing health care among women of reproductive age

After adjusting for individual and community level factors, women's educational status, marital status, occupational status, wealth index, birth in the last years, residence site was significantly associated with problems in accessing health care among women of reproductive age which are presented in Table 6.

**Table 6:** A multilevel mixed effect binary logistic regression analysis of factors associated with problems in accessing health care among women of reproductive age (n = 4680).

Variables	Problems in accessing health care		COR (95%CI)		Model 1 AOR (95% CI)		Model 2 AOR (95% CI)	Model 3 AOR (95% CI)		
	Yes n (%)	No n (%)								
Educational status										
No education	2154 (78.16)	602 (21.84)	5.87 (2.50, 13.82)		2.33 (1.58, 3.45)			2.21 (1.48, 3.30)*		
Primary	856 (66.88)	424 (33.13)	3.33 (1.38, 8.05)		1.67 (1.14, 2.43)			1.58 (1.07–2.32)*		
Secondary	230 (53.12)	203 (46.88)	1.77 (0.65, 4.79)		1.22 (0.82, 1.82)			1.19 (0.79–1.78)		
Higher	81 (38.39)	130 (61.61)	1		1			1		
Marital status										
Married	2435 (72.43)	927 (27.57)	1		1			1		
Not married	886 (67.22)	432 (32.78)	0.80 (0.56, 1.14)		1.30 (1.05, 1.60)			1.30 (1.06–1.59)*		
Occupational status										
Not working	2053 (72.39)	783 (27.61)	2.39 (1.53, 3.75)		1.35 (1.08, 1.70)			1.33 (1.06–1.68)*		
Professional	323 (50)	323 (50.00)	1		1			1		
Agricultural	719 (84.29)	134 (15.71)	4.43 (2.25, 8.72)		2.09 (1.52, 2.89)			1.88 (1.35–2.61)*		
Others	226 (65.51)	119 (34.49)	1.83 (0.87, 3.82)		1.21 (0.87, 1.69)			1.20 (0.86–1.68)		
Religion										
Muslim	2160 (72.07)	837 (27.93)	1		1			1		
Protestant	648 (74.48)	222 (25.52)	1.08 (0.54, 2.12)		1.3 (0.96, 1.84)			1.36 (0.93–1.99)		
Orthodox	428 (60.28)	282 (39.72)	0.55 (0.32, 0.96)		1.15 (0.86, 1.56)			1.17 (0.45–1.63)		
Others	85 (82.52)	18 (17.48)	1.50 (0.26, 8.65)		1.20 (0.62, 2.35)			1.23 (0.61–2.48)		
Household wealth index										
Poor	2377 (81.43)	542 (18.57)	5.34 (3.64, 7.80)		3.45 (2.69, 4.44)			2.95 (2.25–3.86)*		
Middle	281 (72.24)	108 (27.76)	2.74 (1.48, 5.07)		2.00 (1.47, 2.70)			1.74 (1.27–2.40)*		
Rich	663 (48.32)	709 (51.68)	1		1			1		
Head of household										
Male	2137 (71.62)	847 (28.38)	1		1			1		
Female	1184 (69.81)	512 (30.19)	0.86 (0.61, 1.21)		0.88 (0.74, 1.04)			0.90 (0.76–1.07)		
Births in the last five years										
No	1397 (65.99)	720 (34.01)	1		1			1		
One	921 (72.24)	354 (27.76)	1.27 (0.84, 1.93)		1.07 (0.87, 1.31)			1.07 (0.87–1.31)		
Two	795 (78.48)	218 (21.52)	1.63 (1.05, 2.52)		1.30 (1.03, 1.66)			1.29 (1.02–1.64)*		
Three and above	208 (75.64)	67 (24.36)	1.57 (0.81, 3.011)		1.27 (0.89, 1.83)			1.27 (0.88–1.82)		
Residence										
Urban	460 (44.75)	568 (55.25)	1				1		1	
Rural	2861 (78.34)	791 (21.66)	4.41 (2.86, 6.81)				6.11 (4.05, 9.24)		2.16 (1.40–2.02)*	
Region										
Afar	785 (69.59)	343 (30.41)	1				1		1	
Somali	983 (70.67)	408 (29.33)	1.31 (0.78, 2.22)				1.09 (0.68, 1.74)		1.13 (0.73–1.75)	
Benishangul	866 (76.91)	260 (23.09)	1.65 (0.89, 3.04)				1.19 (0.72, 1.96)		1.49 (0.90–2.48)	
Gambela	687 (66.38)	348 (33.62)	0.76 (0.34, 1.70)				0.96 (0.58, 1.58)		1.10 (0.63–1.90)	
Exposure to mass media										
Yes	62 (41.06)	89 (58.94)		1			1			1
No	3259 (71.96)	1270 (28.04)		3.45 (1.27, 9.35)			1.5 (1.03, 2.22)			1.03 (0.69–1.55)

*AOR* adjusted odds ratio, *COR* crude odds ratio, *Model 1* adjusted for individual-level factors, *Model 2* adjusted for community-level factors, *Model 3* adjusted for both individual and community-level factors (full model).

*Statistically significant at p-value < 0.05 in the full model (model 3).

Hence, the odds of experiencing problems in accessing healthcare among women of reproductive age who are uneducated and these who attended primary school was 2.21 (AOR = 2.21 95% CI 1.48–3.30) and 1.58 (AOR = 1.58 95% CI 1.07–2.32) times higher than those who attended higher education, respectively. Women of reproductive age who gave two births in the last five years were 1.29 times higher odds of experiencing problems in accessing healthcare compared to those who did not give birth in the last five years (AOR = 1.29 95% CI 1.02–1.64). The odds of experiencing problems in accessing healthcare among non-married women of reproductive age were 1.30 times higher than married women (AOR = 1.30 95% CI 1.06–1.59). The odds of experiencing problems in accessing healthcare among women of reproductive age who were not working and working in agriculture were 1.33 (AOR = 1.33 95% CI 1.06–1.68) and 1.88 (AOR = 1.88 95% CI 1.35–2.61) times higher than those who do have professional work, respectively. Women of reproductive age who are in the poor and middle household wealth status had 2.95 (AOR = 2.95 95% CI 2.25–3.86) and 1.74 (AOR = 1.74 95% CI 1.27–2.40) times higher odds of experiencing problems in accessing healthcare than those who are in the rich wealth status, respectively. These women of reproductive age reside in the rural area had 2.16 times higher odds of experiencing problems in accessing healthcare than women who reside in the urban area (AOR = 2.16 95% CI 1.40–2.02).

## Discussion

We found that 71.0% of women of reproductive age had at least one serious problem in accessing health care in emerging regions of Ethiopia. Of these, difficulty of obtaining money (57.3%) and distance from health facilities (53.3%) were the most frequently mentioned problems in the regions.

Previous studies in Ethiopia supported our findings as reported problems in accessing health care was 70%^26^ and 72%^37^. Even though the Ethiopian government has made tremendous efforts to decrease the problems in accessing health care for women, our study revealed that there is still a higher magnitude of problems in accessing health care among women of reproductive age in emerging regions of Ethiopia. Moreover, this finding was higher than studies conducted in South Africa 65%^27^, Rwanda 64%^38^, Tanzania 64.5%^23^, and Ghana 51%^39^. The higher finings in our study might be explained due to the study settings; emerging regions (Somali, Afar, Gambela, and Benishangul-Gumuz) were economically disadvantaged and characterized by scattered pastoralist and semi-pastoralist societies which make them to prone to problems in accessing health care in contrast to other regions. In addition, sociocultural, topography and economic differences among countries could be the reason for observed differences.

Our study revealed a significant association between lack of education and difficulties in accessing healthcare. Other studies in Ethiopia^24–26^, South Africa^21^, Ghana^39^, Sub-Saharan Africa^21^, and Tanzania^23^ are also supported our findings. Women of reproductive age who have higher levels of education are more likely to work in higher-paying careers. As a result, they can afford healthcare expenditure regardless of the costs. In addition, they are more aware of their basic human rights and may have better health literacy. Consequently, these educated women of reproductive age are capable to overcoming any type of healthcare challenges than their counterparts, who may also have lower health literacy, which has been identified as a major impediment to health care access^29,40^. This implies that the government should develop special programs that address the education gaps of women who reside in emerging regions of Ethiopia in order to empower and make them independent.

We found that women who are not currently married had higher odds of experiencing problems in accessing health care compared to married women. Our finding is supported with these of the previous studies in Ethiopia^26^, Ghana^39^ and Tanzania^23^. This implies that married women may advance financial and psychological support from their partners to access healthcare^41^. In contrast, studies conducted in Malaysia revealed that married respondents had higher barriers compared to those who are unmarried^42^. The observed difference could be explained due to married women needed permission from their husband or head of household to leave the house or visit health facilities, and their husband may not have given them permission, causing them to have difficulty accessing health care^43,44^.

In our study, women who lived in the rural areas had higher odds of experiencing problems in accessing healthcare than their counterparts. Our finding is supported by previous studies in Ghana^39^, Sub-Saharan Africa^29^, South Africa^27^, Ethiopia^26^, and Tanzania^23^. The explanation could be that rural areas have poor health infrastructure i.e. geographical inaccessibility of health facilities, less privileged and the influences of socio-cultural practices that need women to seek permission from their spouses before visiting healthcare or women's decisions for seeking healthcare services is under their husband/partners control^26^. Moreover, male involvement and support for women health care access are limited^26^. This implies that the government should expand infrastructure services. In addition, efforts should be exerted to encourage male involvement and support for improving health care access for reproductive age women.

In our study, we found that women who are in the poor and middle household wealth status had higher odds of experiencing problems in accessing health care. This finding might be explained by women in the poorest households facing a challenge to cover their basic needs, such as food, and therefore less likely to afford healthcare cost^39,45,46^ and supported by other previous studies in Ethiopia, Tanzania, Egypt, Ghana, and Afghanistan^23,26,28,47,48^. The finding indicates that there is a need to develop strategies and initiatives that safeguard pastoralist inhabitants against drought, flood and social unrest which help to improve their socio-economic status and diminish the barriers that hinder health care access for women of reproductive age.

We found that women who were not working and working in agriculture had higher odds of experiencing problems in accessing health care than those who did have professional work. The finding is supported by other studies in Ghana^39^, Malaysia^49^, Tanzania^23^ and United Kingdom^50^. This could be because women who have good occupations have enough income and independence to afford their health-care costs, allowing them to overcome the health-care access barrier^29^. In contrast, another study conducted in Ethiopia^26^ showed that no significant association between unemployment and problems in accessing healthcare. This could be due to being employed is not enough to have full access to healthcare as there are other problems preventing women from accessing healthcare, such as poor infrastructure, gender inequality, and lack of knowledge regarding health services^23^. The finding implies that it is better to create job opportunities for women who reside in emerging regions to overcome one of the problems in accessing health care; financial hardship.

In our study, to the odds of experiencing problems in accessing healthcare among women who gave two births in the last five years was higher compared to those who had not given birth. This finding is consistent with a study conducted in Sub-Saharan Africa countries^29^. This could be because women have responsibilities for taking care of their children and the household, and due to this, they are unable to leave their homes and become reluctant to seek health care^51^. This implies that the government should establish a strong public health system that is responsible for ensuring that all women who have children have access to primary and preventive health care services.

### Strength and limitations of the study

This study used a nationally representative data with a large sample size as a strength. The study also employed advanced statistical models, which considered the hierarchical nature of the DHS data to get reliable estimates. Moreover, since it is based on the national survey data the study has the potential to give insight for the policymaker and program planners to design appropriate interventions for Pastoralist communities in Ethiopia those lagging in major health indicators.

Despite these strengths, the study finding is interpreted in light of limitations. Supply side predicators of the outcome variables are not included owing to the use of secondary data. Moreover, women might have experienced recall bias, particularly regarding the problems they encountered in the previous five years.

## Conclusions

This study finding revealed that significant proportion of women of reproductive age in emerging region of Ethiopia had experienced problems in accessing health care. This was particularly more pronounced among unmarried women, those in poor and middle quintiles, uneducated and women who attend primary school, women who were not working and working in agriculture, rural residents, and women who gave two births. Even though the government has attempted to incorporate pastoral development in its national development plan (2005–2009), our study finding showed that still great efforts needed to address the identified problems in accessing health care in emerging regions of Ethiopia. Therefore, the government should develop strategies that improve women education, household wealth status, and women occupational status to diminish the barriers that hinder health care access for women living in emerging regions of Ethiopia.

## Data Availability

Data for this study were sourced from Demographic and Health surveys. The database was available at official website of DHS is at https://dhsprogram.com/.
